# Supplementary material for: Are carbon emissions trading and green financial instruments synergistic? -Comprehensive quantitative research based on content analysis
Source: PLoS One. 2024 Mar 7;19(3):e0298601. doi: 10.1371/journal.pone.0298601 (PMC10919601; doi:10.1371/journal.pone.0298601)
Supplement: S1 Data — (ZIP) [file pone.0298601.s001.zip › Data/地级市Z_F工作报告.zip!/╔╜╢1⁄2/╤╠╠¿╩╨/└┤╫╘╣1⁄2╓┌║┼í╛┬φ┐╦╩2╛▌═°í┐_2008.docx]

各位代表：

现在，我代表市人民政府向大会报告工作，请予审议，并请市政协委员和其他列席会议的同志提出意见。

一、过去五年的政府工作

过去的五年是不平凡的五年。面对复杂多变的形势环境和艰巨繁重的发展任务，全市上下在中共烟台市委的正确领导下，坚持以邓小平理论和“三个代表”重要思想为指导，深入贯彻落实科学发展观，按照“又好又快、赶超发展、走在前面”的要求，解放思想，拼搏进取，较好地完成了市十四届人大历次会议确定的任务目标，实现了经济社会发展的新跨越。

过去五年，是经济平稳快速增长、发展质量明显提高、综合实力大幅提升的五年。初步核算，2007年，全市实现生产总值2878.97亿元，是2002年的2.2倍，五年年均增长17.3%。按现行汇率计算，人均生产总值达到6057美元。经济总量在省内和全国首批14个沿海开放城市中实现了位次前移。境内税收收入达到290.4亿元，年均增长23%；地方财政收入达到140.82亿元，年均增长30%。固定资产投资累计完成6524.2亿元，建成和在建总投资亿元以上的项目626个。龙口、莱州、招远、蓬莱四市在全国经济综合实力百强县中位次提升，莱阳市跨入全国县域经济基本竞争力百强县行列。污染排放、单位生产总值能耗逐年下降，土地投资强度提高了4倍。

过去五年，是结构调整取得重大成效、产业优势日益突出、创新能力显著增强的五年。工业四大支柱产业和三大产品集群迅速膨胀，基本形成了集约化、规模化、外向型的现代产业体系。2007年，全市规模以上工业增加值达到1563.58亿元，年均增长32.8%。主营业务收入过百亿元的企业达到5家，新增中国名牌产品和驰名商标37个，高新技术产业产值占规模以上工业的比重由16.5%提高到34.68%。现代服务业繁荣发展，高端服务业蓬勃兴起。服务业实现增加值884.15亿元，年均增长15.4%；社会消费品零售额完成829.7亿元，年均增长16.5%。城市经济、海洋经济发展呈现新局面，占全市经济的比重分别达到41%和14.9%；全市港口货物吞吐量达到1.3亿吨，集装箱125万标箱，进入了全国十大集装箱港口行列。创新型城市建设卓有成效，累计申请国家专利13993件，取得科技成果826项。在第十届中国专利奖评选中，我市3项专利被评为金奖，居全国各城市之首。中科院烟台海岸带可持续发展研究所开工建设，新增1个国家级工程技术研究中心、5个国家级企业技术中心。

过去五年，是体制改革迈出新步伐、对外开放进入新阶段、市场化国际化进程不断加快的五年。社会主义市场经济体制日趋完善，国有资产监管体制进一步健全，市县乡村四级企业改制基本完成，企业公司制、股份制改革深入推进。新增上市公司8家，总数达到16家，五年累计融资68亿元。财税、投资、金融以及社会事业等领域的改革取得新进展。民营经济规模和层次快速提升，实现增加值和上缴税金占全市的比重分别由54.3%和48%提高到78%和67%。对外开放继续向高层次、宽领域方向迈进。累计实际外商直接投资93亿美元，实际利用内资1280亿元，又有15家世界500强企业来我市投资发展。2007年，外贸进出口总额达到239.44亿美元，其中出口140.92亿美元，均为2002年的5.3倍，经济外向度达到60%。机电产品和高新技术产品出口比重分别达到55%和23%，欧美高价位市场出口份额达到52%。我市西北林业公司承建的中俄-托姆斯克木材工贸合作区成为全国首批11个国家级对外经贸合作区之一。经济技术开发区在全国54个国家级园区中综合评价由第14位上升到第6位，出口加工区实现功能升级，新建了栖霞台湾农民创业园和3个省级开发区、7个国别工业园区。与5个国外城市缔结了友城关系。成功举办了首届烟台国际葡萄酒节、国际果蔬·食品博览会、APEC及亚欧会议系列经贸活动，取得了丰硕成果。

过去五年，是大城市框架基本形成、基础设施日臻完善、城市面貌发生深刻变化的五年。中心城市实现了“五年大变样”的目标。完成了新一轮城市总体规划修编。打通了塔山、魁星楼、黄金顶、通世南路4条隧道，建设改造了滨海路、连城路等80多条城市道路，拉开了大市区发展框架，建成区面积由137平方公里扩大到176平方公里。新建了体育公园、国际博览中心、滨海景区，启动了大南山生态景区开发，综合整治了南大街和54个社区，升级改造了部分垃圾和污水处理厂，加大了绿化、亮化、净化、美化力度，市区13万多户居民用上了天然气，城市环境质量和宜居水平明显提高。县级城市和小城镇发展步伐加快，全市城市化水平由48%提高到54.6%。烟大铁路轮渡、威乌高速和威青高速烟台段以及蓝烟铁路复线、大莱龙铁路投入使用，引黄调水、烟台港西港区、龙烟铁路、火车站改造、机场改扩建等重大项目进展顺利，海阳核电已经进入主体工程建设阶段。

过去五年，是支农惠农力度不断加大、农村生产生活条件持续改善、新农村建设扎实推进的五年。农业产业化、国际化、标准化水平逐步提高。新增销售收入过亿元的农业产业化龙头企业48家。2007年，全市农产品出口达到24.5亿美元，年均增长21.8%。烟台苹果、龙口粉丝、烟台大樱桃和烟台葡萄酒成为国家地理标志保护产品。扶农惠农政策全面落实，农业税全部免除。新型农村合作医疗普遍推开，参合率达到93.4%。大力实施农村劳动力转移培训阳光工程，转移农村富余劳动力18万人。完成了农村电网建设改造工程和1000个村的“四清四化”整治任务，农村公路硬化通村率、自来水普及率、有线电视入户率分别达到97%、96.1%和91%。

过去五年，是各项事业繁荣发展、群众得到更多实惠、社会和谐稳定的五年。义务教育、素质教育、高中段教育水平不断提高，高等教育快速发展，新增8所高校。在全省率先免除了城乡义务教育阶段学生学杂费，建立了家庭困难学生资助机制。覆盖城乡的疾病防控体系基本形成，取得了抗击“非典”和防控“禽流感”的重大胜利。产品质量和食品药品安全监管力度加大。文化事业和文化产业繁荣发展，精神文明建设进一步加强。成功承办了第二十一届省运会和第七届省残运会，在第二十八届奥运会等国内外重大赛事上，我市运动员均取得优异成绩。人民生活水平显著提高，城市居民人均可支配收入和农民人均纯收入分别达到16772元和6979元，年均增长13.4%和12.7%。居民消费价格总指数涨幅控制在3.5%以内。就业再就业工作富有成效，五年新增城镇就业再就业61.7万人，城镇登记失业率控制在3.4%以内。社会保障工作进一步加强，按时足额为各类保障对象支付社会保险待遇167亿元，五保对象集中供养率保持在80%以上。市中心区累计供应经济适用房3700套，1075户住房困难家庭享受到廉租房待遇。爱心捐助活动累计捐款4.2亿元，救助困难群众70多万人次。高度重视信访工作，提升市长公开电话功能，每年组织实施为民服务实事。应急管理和安全生产管理不断加强，社会治安持续稳定。全市森林覆盖率达到37%。人口连续9年实现“零增长”。

帮扶栖霞、支持长岛和支援定陶、聂拉木等工作取得实效。老龄、妇女、儿童、残疾人、红十字会以及广播电视、新闻出版、气象地震、史志档案、双拥共建、人民防空、民族宗教、仲裁等工作也都取得了新的成绩。

过去五年,是民主法制建设全面加强、政府职能加快转变、发展环境进一步优化的五年。基层群众自治机制逐步完善,人民群众的民主权利依法得到保障。实施了“五五”普法依法治理规划,全社会法制意识明显增强。各级政府自觉履行职能,认真办理人大代表建议、政协提案,主动接受人大、政协、民主党派和社会各界的监督,严格落实政务公开和民主决策制度,深化行政管理体制改革,推行行政执法责任制,法治政府建设纵深推进。深入开展“改进机关作风、优化政务环境”活动,加大行风评议和纠风专项治理力度,加强执法监察、行政效能监察和审计监督,深化行政审批制度改革,提高政务服务质量,增强了政府的公信力和执行力。全面加强廉政建设,维护了政府机关和公务员队伍的良好形象。

各位代表,过去五年,我们在科学发展的道路上迈出了坚实步伐,从经济到社会、从城市到乡村、从物质生活到精神面貌都发生了巨大变化,得到了各级领导、社会各界和人民群众的充分肯定和广泛认同。五年来,我市获得了全国文明城市、最佳中国魅力城市、全国社会治安综合治理优秀城市、全国双拥模范城市、国家环保模范城市、国家园林城市、中国优秀旅游城市、中国投资环境金牌城市、全国创新环境十强城市、全国生活质量十强城市以及中国人居环境奖、联合国人居奖、联合国迪拜改善居住环境最佳范例奖等一大批荣誉,城市知名度和影响力空前提高,广大群众的自豪感和荣誉感显著增强。

这些成绩,是全市人民在上级党委、政府和市委的正确领导下,团结拼搏、努力进取的结果;是人大、政协及各民主党派依法监督、大力支持的结果;是社会各界和海内外友好人士热心关注、积极参与的结果。在此,我代表市人民政府,向全市广大工人、农民、知识分子以及各阶层的建设者,向驻烟人民解放军、武警部队官兵和政法干警,向中央、省属驻烟单位,向各位人大代表和政协委员,向各民主党派、工商联、无党派人士和各社会团体,向所有关心支持烟台发展的香港和澳门特别行政区同胞、台湾同胞、海外侨胞以及国际友人,表示衷心的感谢!

各位代表,我市经济社会发展取得了很大成绩,但前进中还面临不少困难和问题,主要是:经济发展方式还比较粗放,结构性矛盾仍然突出,自主创新能力不强,农业稳定发展和农民持续增收压力加大,服务业发展相对滞后,节能减排和环境保护任务艰巨;对外开放层次还不够高,一些领域的改革有待于深化;城市建设发展水平还需进一步提升;经济与社会、城市与农村以及区域间发展不够平衡,就业、保障、教育、卫生、住房、分配等关系群众切身利益的问题解决得还不够好;政府工作还不能很好地适应新形势新任务的要求,官僚主义、形式主义和消极腐败现象不同程度地存在,等等。这些问题,我们一定高度重视,认真加以解决。

各位代表,回顾五年来的探索与实践,我们深刻体会到,加快推进改革开放和现代化建设,不断开创我市经济社会发展新局面,必须始终把解放思想作为一大法宝,高举中国特色社会主义伟大旗帜,与时俱进,开拓创新,积极探索符合烟台实际的发展道路;必须始终把发展作为第一要务,紧紧抓住经济建设这个中心,以科学发展观统领全局,坚持好字优先、又好又快,努力转变发展方式,实现科学、和谐、率先发展;必须始终把改革开放作为发展的最大动力,毫不动摇地推进体制机制创新,坚定不移地实施经济国际化战略,用开放的思维和改革的办法解决前进中的困难和问题;必须始终把实现好、维护好、发展好人民群众的根本利益作为一切工作的出发点和落脚点,坚持以人为本、富民优先,高度重视以保障和改善民生为重点的社会建设;必须始终把统筹兼顾作为工作的基本方法,自觉转变工作指导方式,正确处理改革发展稳定的关系,维护公平正义,促进社会和谐;必须始终把坚持党的领导作为推进各项事业的根本保证,坚决贯彻执行党的路线方针政策,认真落实市委决策部署,充分调动全市上下的积极性、创造性,形成推动发展的强大合力。这些经验体会,是我们的宝贵财富,我们要倍加珍惜,不断发扬,以更加积极的姿态投入到现代化建设的伟大实践当中,在科学发展的道路上阔步前进!

二、今后五年的发展任务

各位代表,从现在到2012年的五年,是我市贯彻落实党的十七大精神、推动经济社会科学发展的重要时期,也是站在新起点、再创新优势,全面建设小康社会的关键时期。我们必须适应国内外形势的新变化,顺应全市人民过上更好生活的新期待,把握经济社会发展趋势和规律,站在时代的高度,用世界的眼光谋划未来,推动发展,谱写改革开放和现代化建设的新篇章。未来五年,我们要全面贯彻党的十七大、省九次党代会和市十一次党代会精神,高举中国特色社会主义伟大旗帜,以科学发展观为统领,坚持稳中求进、好字优先、又好又快,着力转变工作指导和发展方式,着力提高经济综合实力和运行质量,着力推进改革开放和自主创新,着力加强资源节约和环境保护,着力促进社会发展和解决民生问题,加快工业化、城市化、市场化和国际化步伐,推动经济社会科学、和谐、率先发展,全面建设文明富庶新烟台。工作中,努力实现以下目标:

———经济更加繁荣。抓住省委省政府实施“一体两翼”区域发展战略、国家推进天津滨海新区开发和振兴东北老工业基地等重大机遇,拓展发展空间,培育发展优势,实现综合实力的新突破。大力推进经济结构战略性调整,壮大先进制造业支柱产业,打造现代服务业优势板块,提升现代农业发展层次,培育高新技术产业集群,建立富有竞争力的现代产业体系。进一步转变经济发展方式,更加注重科技进步、劳动者素质提高和管理创新,更加注重消费、投资、出口的协调拉动和三次产业的协同带动,提高经济运行质量和可持续发展能力。继续保持经济平稳较快增长,主要经济指标增幅高于全省和全国首批14个沿海开放城市平均水平。到2012年,全市生产总值在2007年的基础上再翻一番,达到5800亿元;三次产业比例调整到4:58:38;全市境内税收收入达到600亿元,年均增长16%;地方财政收入达到320亿元,年均增长18%;高新技术产业产值占规模以上工业的比重达到40%以上。

———发展更具活力。不断解放思想,坚持与时俱进,形成与科学发展相适应的思维方式、发展理念,营造加压奋进、竞相发展的浓厚氛围。推进市场取向改革,构建充满活力、富有效率、更加开放、有利于科学发展的体制机制。全面提高开放水平,完善内外联动、互利共赢、安全高效的开放型经济体系,形成经济全球化条件下参与国际经济合作和竞争的新优势。大力推进自主创新,建立以企业为主体、市场为导向、产学研相结合的技术创新体系,实施知识产权战略,加快建设创新型城市。到2012年,民营经济增加值占全市生产总值的比重达到80%以上;全市外贸进出口总额达到500亿美元,实际外商直接投资年均增长10%,经济外向度达到65%;科技进步对经济增长的贡献率达到60%。

———城乡更加秀美。加快中心城市建设,按照大城市、大市区的发展要求,完善发展框架,增强承载功能,彰显城市特色,向着现代都市的方向快速迈进。加快县级城市和小城镇建设,增强聚集辐射能力,强化对周边的带动作用,提高城市化水平。加快新农村建设,建立以工补农、以城带乡、财政支农长效机制,实现城乡统筹发展,共同繁荣。加快基础设施建设,解决好交通、能源、水资源等方面的制约因素,构筑强有力的支撑保障体系。加快生态市建设,形成符合生态文明要求的产业结构、增长方式和消费模式,加强水土保持和生态修复,构建资源节约型、环境友好型社会。到2012年,中心城市基本实现五区融合,建成区面积达到210平方公里,人口达到200万;全市城市化水平达到60%;森林覆盖率达到40%。

———社会更加和谐。加强社会主义民主法制建设,保障人民依法行使民主权利。推进依法治市,建立起科学规范的行政执法体制。完善社会服务管理体系,增强公共服务能力。打造烟台特色文化,加强社会主义核心价值体系建设,推动社会文明进步。坚持改革发展稳定相协调,及时预防化解各种矛盾,保障社会公平正义,创建和谐稳定模范城市,努力形成全市人民各尽其能、各得其所而又和谐相处的局面。

———人民更加幸福。坚持以人为本,促进人的全面发展,做到发展为了人民、发展依靠人民、发展成果由人民共享。不断增加公共财政对保障和改善民生的支出,解决好劳动就业、收入分配、社会保障、文化教育、医疗卫生、居民住房和困难群体生活等问题,使全市人民学有所教、劳有所得、病有所医、老有所养、住有所居。城市居民人均可支配收入和农民人均纯收入年均分别增长9%和8%以上,城镇登记失业率控制在3.3%以内,城乡人均住宅建筑面积分别达到30和35平方米以上。

各位代表,经过五年的努力,我们生活的这个城市将进一步发展成为一个产业体系先进、综合实力雄厚、区域中心地位突出的经济强市,成为一个社会和谐稳定、人民安居乐业、文化特色鲜明的文明之都,成为一个功能完备、山川秀丽、富有魅力的宜居名城。我们坚信,有中共烟台市委的正确领导,有市人大、政协的监督支持,有历届市政府奠定的良好基础,全市人民团结一心、埋头苦干,改革发展的各项任务一定能够完成,烟台的明天一定会更加美好!

三、2008年政府工作安排

2008年是全面贯彻落实党的十七大精神的第一年,也是新一届政府工作的起步年,做好今年的工作意义重大。当前,环境形势复杂多变,机遇挑战交织并存,我们必须保持清醒头脑,增强忧患意识,坚定发展信心,牢牢把握机遇,妥善应对挑战,努力推动经济社会又好又快发展,为全面完成未来五年的任务目标打下坚实基础。2008年,全市经济社会发展主要预期目标为:生产总值增长14%;地方财政收入增长20%;规模以上工业增加值增长18%;固定资产投资增长18%;社会消费品零售额增长16%;实际外商直接投资增长12%,外贸进出口总额和外贸出口均增长17%;城市居民人均可支配收入增长10%,农民人均纯收入增长9%;城镇登记失业率控制在3.1%以内;物价涨幅控制在4%以内;人口自然增长率控制在2.4‰以内;万元生产总值能耗下降4.5%,二氧化硫和化学需氧量排放分别减少4%和6%。

(一)推动自主创新,加强节能环保,转变经济发展方式。把提高自主创新能力作为推动科学发展的核心战略,进一步完善技术创新、知识创新和创新服务体系,抓好中科院烟台海岸带可持续发展研究所和北大、清华、烟大“三校科技园”及留学人员创业园区建设,创建1个国家级企业技术中心,取得一批高水平科技成果。促进信息化与工业化融合,加快发展高新技术产业。加大节能减排攻坚力度。加快淘汰水泥、电力等重点行业落后生产能力,加强110户重点用能企业节能管理,培育10个循环经济产业链条,搞好重点燃煤电厂脱硫工程,加速推进污水处理、雨污分流等工程项目建设,完善重点污染源自动监控系统。强化节能环保执法,实行工业园区环境影响评价,严肃查处影响经济发展、危害群众环境利益的违法行为。更加注重集约用地,整顿规范土地市场,依法回收闲置土地,搞好土地储备。加强和改进投资工作,严格执行国家宏观调控政策,引导资金投向高技术含量、高附加值、低能耗、低污染产业,进一步优化投资结构。继续开展“项目推进年”活动,突出抓好110个总投资2987亿元的重点项目。

(二)膨胀产业规模,增强竞争实力,加快建设先进制造业基地。发展壮大产业集群。充分发挥资源、产业优势,积极引进和培植一批重大产业项目,发展终端产品,促进产业聚集,拉长产业链条,提升产业素质。继续膨胀机械、电子、食品、黄金四大支柱产业和汽车、手机、电脑三大产品集群,加快培育核电、石化、冶金、生物制药、葡萄酒等潜力产业和船舶、特种化纤等新的产品集群。抓住建设海阳核电的机遇,引进发展一批核电配套设备、核电技术服务等项目,尽快使核电产业成为新的支柱产业。规划建设石化产业园,引进具有全局拉动作用的战略项目,推动石化产业快速崛起。培强做大骨干企业。继续实施“3·50工程”,落实各项支持奖励政策,推动重点企业与国内外大企业、大财团开展战略合作,培育一批技术水平高、带动能力强、竞争实力雄厚的大型骨干企业。力争主营业务收入过50亿元的企业达到15家,其中过百亿元的企业达到7家。大力实施品牌战略。引导企业提高产品质量,搞好品牌的策划、经营和保护。力争再创3个中国名牌产品和中国驰名商标。

(三)打造优势板块,扩大消费需求,繁荣发展现代服务业。加强重点城区、园区、企业等服务业载体建设,形成一批特色突出、优势明显的服务业聚集区,提高服务业规模化、产业化水平。优化服务业行业结构。积极发展商贸餐饮业,抓好世界广场、佳世客购物中心、阳光100城市广场等市区十大商贸项目和一批美食城、餐饮街建设。加快发展现代物流业,启动建设6个临港物流园区,扶持发展骨干物流企业,构筑功能齐全的现代物流服务体系。大力发展旅游会展业,推进大南山生态景区、雨岱山渔人码头和旅游大世界建设,构筑以中心城市为重点的“1+3”旅游主体板块,高标准办好第二届烟台国际葡萄酒节和第十届国际果蔬·食品博览会,打造知名会展品牌。有序发展金融保险业,规划建设烟台金融中心,引进中外金融机构,增强恒丰银行、城市商业银行和农村合作金融实力,优化金融生态环境。规范发展房地产业,加强房地产市场调控,调整土地供给,建立合理的住房供应体系。扶持发展服务外包业,抓好烟台市服务外包基地、国家动漫产业发展基地和烟台软件园建设。繁荣发展文化产业,培育以现代传媒、印刷出版、文化娱乐为主体的文化产业群体。活跃夜间经济和节假日经济,扩大社区服务需求,推进“万村千乡市场工程”,优化消费环境,培育消费热点,推动消费升级。服务业增加值完成1050亿元,增长17%。

(四)发展农村经济,改善农村环境,全面建设社会主义新农村。调整优化农业产业结构,稳定粮油生产,发展高效生态农业、开放型农业和品牌农业,推进农业标准化生产和产业化经营。新认证无公害农产品、绿色食品和有机食品30个,新发展农业标准化基地50万亩、畜牧标准化养殖场1000个,新增销售收入过亿元的龙头企业8家。抓好病险水库除险加固、节水灌溉等水利工程,实施为期三年的造林工程,提高农业机械化水平,搞好重大动物疫病防控工作。稳妥推进农村土地流转,提高农村土地规模经营水平和产出效益。积极探索“一镇一业、一村一品”的发展路子,扶持发展农村特色产业,拓宽农民增收渠道。实施新型农民培训工程,提高农民就业能力,新增农村劳动力转移就业2万人以上。完善农村公共服务体系,推动公共财政向农村倾斜、基础设施向农村延伸、社会保障向农村覆盖,加快实现城乡基本公共服务均等化。认真落实各项支农惠农政策,促进农业增效、农民增收、农村发展。组织实施"示范村"创建活动,加大“四清四化”整治和电气化村镇建设力度,农村公路硬化通村率达到98%。深入开展“3+1”联手共建活动,形成全社会共同参与新农村建设的生动局面。

(五)完善承载功能,繁荣城市经济,强化中心城市辐射带动能力。实施城市总体规划、海岸带规划和“十一五”城市近期建设规划,尽快实现城乡规划全覆盖。完成火车站改造、机场改扩建、烟台至莱州沿海大通道主体工程、滨海东路延伸、双河东西路、德龙烟铁路“两隧一桥”、引黄调水控制性建筑和输水明渠等建设任务,增强基础设施承载功能。组织好莱山中心区片开发建设,加快扩展新区规模。实施幸福区片综合整治、北马路拓宽改造、朝阳街和所城里等历史街区保护性改造、旧居民楼“平改坡”等一批老城区改造项目,抓好过街通道、城市公园、停车场等公益性项目建设,方便群众生活,提升城市形象。进一步理顺管理体制,优化公用设施网络,加快五区一体化进程。推行城市数字化管理,提高管理效能。优先发展城市公交,改善城市交通状况。按照“扩大规模、提升素质、增强活力”的思路,加快发展城市经济,提高开发区-福山板块的现代制造业水平,膨胀芝罘-莱山板块的现代服务业规模,加大牟平板块的综合开发力度,推动中心城市经济向高端化发展。

(六)拓展发展领域,挖掘发展潜力,培育经济增长新优势。合理开发海洋资源,大力发展海洋经济和临港产业。实施“以港兴市”战略,重点推进烟台港西港区一期工程、芝罘湾港区集装箱码头、龙口港区通用泊位工程建设。加快建设临港产业带,积极发展大型船舶、海洋工程船舶等装备制造业。繁荣壮大海洋运输和海洋渔业,着力培育海洋化工、海洋能源、海洋服务等海洋新兴产业。全市港口货物吞吐量达到1.4亿吨,集装箱吞吐量达到140万标箱;海洋产业产值增长16%,临港工业产值增长20%以上。全面落实扶持政策,活跃发展民营经济。实施小企业培育、中小企业成长和特色产业提升“三项计划”,加大专业村、特色镇、产业密集区培育力度,营造关爱企业家、尊重纳税人、支持创业者的社会氛围。新发展个体工商户和私营小企业2万家以上,新增规模以上民营企业300家以上,主营业务收入过10亿元的企业达到40家以上,民营经济增加值增长16%。进一步激发县域经济发展活力,逐步形成功能定位清晰、发展导向明确、开发秩序规范的发展格局,鼓励强市率先发展,支持中等市跨越发展,扶持困难县市赶超发展,促进各县市在全国、全省县域经济中位次前移。

(七)扩大引资规模,调整外贸结构,提高开放型经济水平。创新招商方式和引资理念,优化利用外资结构,引进世界500强、跨国公司和国内知名企业来烟投资,建立生产基地、研发中心、营运中心和区域总部,引导外资投向先进制造业、高端服务业、现代农业、城市基础设施和社会事业。转变外贸增长方式,促进机电产品和高新技术产品出口,扩大短缺资源、先进技术、关键装备产品进口,加快加工贸易转型升级。机电产品和高新技术产品出口比重分别达到60%和25%。培育骨干出口企业,推动10家企业进入国家和省级出口名牌行列,2家企业成为国家和省级科技兴贸出口创新基地。积极实施“走出去”战略,鼓励企业开发境外资源,支持有品牌优势和自主知识产权的企业到境外建立生产和营销基地,扩大劳务外派与境外工程承包。充分发挥外事、侨务、贸促、对台、口岸工作在推进对外开放中的优势和作用。进一步加强园区建设,增强经济技术开发区龙头带动作用,拓展出口加工区综合服务功能,推进栖霞台湾农民创业园建设,提升各类园区的发展层次。建设国际产业转移配套社区一期工程,完善大通关协作机制,进一步优化开放环境。

(八)深化各项改革,创新体制机制,增强发展的动力和活力。调整优化国有经济布局,强化国有资产监管,提高国有经济支撑力和影响力。积极培植企业上市后备资源,加强企业上市工作。整合技术交易服务机构,促进科技成果转化和高新技术企业产权流动。做好新一轮行政管理体制和机构改革准备工作,推进事业单位改革。创新财政管理,强化财源建设和税收征管,优化财政支出结构,扩大公共财政覆盖范围,完善政府集中采购办法,加强财政监管,保证各级财政健康运行。健全价格管理和价格形成机制,保持物价稳定。深化医疗卫生、义务教育和城市公用事业领域改革。

(九)发展社会事业,加强社会建设,巩固稳定和谐的良好局面。继续提高和均衡发展义务教育,积极推进素质教育,大力发展职业技术教育和高等教育,支持驻烟高校发展,抓好烟台职业学院和汽车工程职业学院建设,完善义务教育经费保障机制。实施引才、育才、聚才工程,建立与科学发展相适应的人才队伍。搞好市文化中心、广电中心建设,完善县乡村三级文化设施,推进广电“三通”、文化信息资源共享、农村书屋工程,加快广播电视数字化进程,构建覆盖全社会的公共文化服务体系,更好地满足人民群众文化生活需求。组织好文化遗产保护、文物普查和古籍保护工作。开展全民健身活动,提高竞技体育水平,认真做好第三届亚洲沙滩运动会和第十一届全运会烟台赛区的筹备工作,办好烟台市第七届运动会。加强人口和计划生育工作,稳定低生育水平,提高出生人口素质。发展残疾人和老龄事业,完成500户农村贫困残疾人危房改造,巩固建设100处残疾人就业扶贫基地、100个农村“老年之家”示范点。抓好妇女儿童、外事侨务、新闻出版、气象地震、史志档案、人民防空、民族宗教、仲裁、红十字会以及国防教育、国防动员、民兵预备役、双拥共建工作。全面加强精神文明建设,广泛深入开展思想道德建设活动,弘扬“创新、拼搏、协作、包容”的烟台精神,把社会主义核心价值体系融入全民教育的全过程。加强人民来信来访工作,完善社会矛盾纠纷调解机制,杜绝在企业改制、土地征用、房屋拆迁中侵害群众利益的现象。加大食品药品专项整治力度,强化源头监控和市场监管,保障饮食用药安全。进一步提高应急管理能力,加大安全生产监管、执法检查和综合整治力度,健全社会治安防控体系,严厉打击各类违法犯罪活动,为改革开放和现代化建设创造优良的社会环境。

(十)坚持以人为本,改善民生质量,认真解决关系群众切身利益的实际问题。实施扩大就业的发展战略,建设市级人才和人力资源市场,完成统筹城乡就业试点任务。年内新增城镇就业再就业13万人以上,基本消除城镇“零就业”家庭和农村“零转移就业贫困家庭”,大中专毕业生总体就业率达到75%以上。建立企业职工工资正常增长机制和支付保障机制,落实农民工有关政策,依法维护劳动者权益。健全完善社会保障救助体系。进一步扩大社会保险覆盖面,做实企业职工基本养老保险个人账户。全面推行城镇居民基本医疗保险制度,城镇非从业居民参保率达到60%以上。健全农村五保供养体制。推行新型农村社会养老保险制度和被征地农民社会保障实施办法。扩大低保覆盖范围,提高城乡低保标准,农村低保对象年人均补差500元以上。继续开展爱心捐助活动。搞好粮油等重要物资储备,建立物价上涨与提高困难群众生活补贴和保障标准联动机制,保证群众基本生活质量。加强住房保障工作,将廉租住房保障范围扩大到低收入住房困难家庭,市中心区新建保障性住房不少于3000套、24万平方米。免除农村义务教育阶段学生课本费,建立完善各类学生资助政策体系,不让一个孩子因贫失学。加强疾病预防控制,建设公共卫生应急机制和医疗急救体系,完善农村三级医疗服务网络和新型农村合作医疗制度,发展社区卫生服务,强化城乡医疗救助和惠民医疗措施。建立健全保障和改善民生的长效机制,切实解决好广大群众最关心、最直接、最现实的利益问题,让全市人民共享发展成果。

四、切实加强政府自身建设

面对新形势新任务,各级政府必须加快转变职能,改进工作作风,不断提高依法行政、民主行政、科学行政的能力和水平,努力建设为民、务实、清廉政府。

(一)提升思想境界,增强推动科学发展能力。认真学习贯彻党的十七大精神,深入领会科学发展观的内涵和实质,增强贯彻落实科学发展观的自觉性和坚定性,提高推动科学发展的思想水平,把握发展规律,创新发展理念,转变发展方式,破解发展难题,提高发展质量。坚持高点定位,不断加压奋进,聚精会神搞建设,一心一意谋发展,努力实现“走在前面”的目标要求。倡导刻苦学习、学以致用的良好风气,提高理论素养、知识水平和创新能力,增强政府机关和公务员队伍的综合素质,开创各项工作新局面。

(二)改进公共管理,优化政务服务。按照社会主义市场经济体制的要求,进一步转变政府职能,调整优化政府职责分工,形成权责一致、分工合理、执行顺畅、监督有力的行政管理体制。完善重大事项集体决策、专家咨询、社会公示与听证相结合的决策机制,提高政府决策民主化、科学化水平。扩大政务公开范围,健全新闻发布制度,加强电子政务和政府网站建设,保障人民的知情权、参与权、表达权和监督权。规范和精简行政审批,严肃查处违规收费、检查、评比和“吃、拿、卡、要”行为,完善政府机关工作规则和服务标准,提高政务服务质量。

(三)加强法制建设,提高依法行政水平。深化行政执法体制改革,强化行政复议监督,健全执法过错责任追究制度和执法行为评议考核制度,加强执法监督和行政监察,规范行政行为,保证各级行政机关及其工作人员严格按照法定权限和程序行使职权、履行职责。坚持向人大及其常委会报告工作和向人民政协通报情况制度,自觉接受市人大及其常委会的法律监督和市政协的民主监督,接受群众监督和舆论监督,高度重视人大代表建议和政协提案办理工作。

(四)转变工作作风,弘扬实干精神。坚持办实事、求实效,集中精力推动工作落实。坚决反对形式主义、官僚主义,反对弄虚作假、急功近利,反对盲目攀比、奢侈浪费。大兴求真务实之风,发扬艰苦奋斗、埋头苦干精神,深入基层开展调查研究,及时解决改革发展稳定中遇到的新矛盾、新问题。进一步强化执行能力,健全完善决策目标、执行责任、考核监督三个体系,解决好"懒、散、推、拖"问题,确保政令畅通。坚持立党为公、执政为民,把人民群众满意作为政府工作的最高标准,倾听群众呼声,关心群众疾苦,做到权为民所用、情为民所系、利为民所谋。

(五)强化廉政建设,维护政府良好形象。把廉政建设和反腐败斗争作为一项重大政治任务,坚持"标本兼治、综合治理、惩防并举、注重预防"的方针,从源头上预防和解决腐败问题。认真落实党员领导干部廉政建设责任制,加大对领导干部和权力部门、重点行业的监督力度,从严防范惩治公务人员失职渎职行为和职务犯罪行为。各级政府领导干部要以身作则、率先垂范,广大公务员要严于律己、克己奉公,正确行使人民赋予的权力,以反腐倡廉的实际行动赢得人民群众的信任。

各位代表,回顾过去,成绩令人鼓舞;展望未来,我们信心百倍。让我们紧密团结在以胡锦涛同志为总书记的党中央周围,在中共烟台市委的坚强领导下,高举中国特色社会主义伟大旗帜,团结和依靠全市广大干部群众,解放思想、开拓创新,同心同德、奋发进取,为全面建设文明富庶新烟台而努力奋斗!
